# Predictive performance of aortic arch calcification for clinical outcomes in patients with acute coronary syndrome that undergo percutaneous coronary intervention: A prospective clinical study

**DOI:** 10.1097/MD.0000000000018187

**Published:** 2019-11-27

**Authors:** Xiaoteng Ma, Lisha Dong, Qiaoyu Shao, Zhen Zhou, Jing Tian, Yue Ma, Jie Yang, Sai Lv, Yujing Cheng, Hua Shen, Lixia Yang, Zhijian Wang, Yujie Zhou

**Affiliations:** a Department of Cardiology, Beijing Anzhen Hospital, Capital Medical University, Beijing Institute of Heart Lung and Blood Vessel Disease, Beijing Key Laboratory of Precision Medicine of Coronary Atherosclerotic Disease, Clinical Center for Coronary Heart Disease; b Department of Radiology; c Department of Nuclear Medicine, Beijing Anzhen Hospital, Capital Medical University, Beijing, China.

**Keywords:** acute coronary syndrome, aortic arch calcification, chest x-ray, clinical outcomes, percutaneous coronary intervention

## Abstract

Currently, little is known regarding the predictive utility of aortic arch calcification (AAC) for clinical outcomes in patients with acute coronary syndrome (ACS) who undergo percutaneous coronary intervention (PCI). The present study was designed to investigate the predictive performance of AAC as detected by chest x-ray for clinical outcomes among ACS patients undergoing PCI.

A total of 912 patients who were diagnosed as ACS and treated with PCI were included in this prospective, cohort study. All study participants received chest x-rays on admission, and a semiquantitative 4-point scale was used to assess the extent of AAC. The primary end point was defined as a composite of major adverse cardiovascular events (MACE) comprising death, nonfatal stroke, nonfatal myocardial infarction, and unplanned repeat revascularization. The key secondary end point was the composite of cardiovascular death, nonfatal stroke, and nonfatal myocardial infarction. The prognostic values of AAC were assessed in multivariate Cox-proportional hazards regression analyses adjusted for major confounders.

The mean follow-up duration was 917 days and, during the follow-up period, MACE occurred in 168 (18.4%) patients. Kaplan-Meier analyses revealed significantly higher incidences of the primary and key secondary end points in patients with higher AAC grades (log-rank test; all *P* < .001). Multivariate Cox-proportional hazards regression analyses showed that, in comparison to AAC grade 0, the hazard ratios of AAC grades 1, 2, and 3 for predicting MACE were 1.63 (95% confidence interval [CI] 0.99–2.67), 2.15 (95% CI 1.27–3.62), and 2.88 (95% CI 1.41–5.86), respectively. The C-index of the variables, including peripheral arterial disease and serum levels of triglyceride for predicting MACE, was 0.644 (95% CI 0.600–0.687) versus 0.677 (95% CI 0.635–0.719) when AAC grades were also included; the continuous net reclassification improvement was 16.5% (8.7%–23.4%; *P* < .001).

The extent of AAC as detected by chest x-ray is an independent predictor of MACE among ACS patients undergoing PCI. Further research is warranted to evaluate whether specific treatment strategies that are established based on AAC extent are needed for optimal risk reduction in relevant patient populations.

## 1 Introduction

Acute coronary syndrome (ACS) presents a wide spectrum of risks for adverse cardiovascular (CV) outcomes for affected patients. Despite widespread use of primary or elective percutaneous coronary intervention (PCI), patients diagnosed with ACS have an increased short- and long-term risk of adverse CV events.^[1]^ Early risk stratification is crucial in assessing prognosis and for guiding optimal treatment of secondary prevention in patients with ACS who undergo PCI.

Arterial calcification has long been considered to be a complication of advanced atherosclerosis.^[2]^ Calcifications in the thoracic aorta are associated with similar risk factors as coronary atherosclerosis,^[3,4]^ which implies that thoracic aortic calcification and coronary atherosclerosis share common underlying systemic vascular atherosclerotic mechanisms. The aortic arch has been identified to be the most vulnerable for calcification in the thoracic aorta^[5]^ and the degree of calcification in the aortic arch can be used to precisely evaluate the magnitude of calcification in the whole aorta.^[6,7]^ A recent study reported that aortic arch calcification (AAC), as determined by computed tomography, has an even better predictive value for mortality than calcification of coronary arteries, as well as extracranial and intracranial internal carotid arteries.^[8]^ Computed tomography has been recognized as the criterion standard for evaluating arterial calcification, yet it is expensive and cannot be easily or commonly performed for routine clinical practice.^[9]^ Chest x-ray techniques are the most widely performed radiological study in patients during hospital stays and can provide relevant and reliable information on AAC.^[10]^ In fact, AAC that is detected by chest x-ray has been demonstrated to be an independent risk factor for CV morbidity and mortality in the general population and several patient groups.^[3,9,11–15]^ In light of these available data, we hypothesize that AAC detected by chest x-ray might be a good candidate for risk stratification for ACS patients undergoing PCI. However, there are insufficient studies regarding the association between AAC and clinical outcomes among ACS patients undergoing PCI.

In the present study, we aimed to prospectively investigate the role of AAC as detected by chest x-ray for predicting adverse CV events among ACS patients undergoing PCI.

## 2 Materials and methods

### 2.1 Study population

A total of 998 patients were admitted to our CV center and diagnosed with ACS, followed by treatment with primary or elective PCI from June 2016 to March 2017. These patients consecutively screened for participation in this single-center, prospective, cohort study and ACS was diagnosed according to the American College of Cardiology/American Heart Association guidelines.^[16,17]^ We excluded patients with previous coronary artery bypass grafting, renal dysfunction with creatinine clearance (CrCl) <30 mL/min, Killip class >2, left ventricular ejection fraction (LVEF) <30%, and any known aortic diseases, such as aortitis, aortic aneurysm, or dissection. Patients in whom the chest x-ray image quality was not sufficient for interpretation were excluded. Two patients were excluded because of missing follow-up data despite at least 4 separate attempts to contact them. Thus, 912 patients were included in the final analysis with a mean follow-up duration of 917 days.

The present study was performed in accordance with the Helsinki Declaration of Human Rights. The study was approved by the local ethics committee of Beijing Anzhen Hospital, Capital Medical University. All patients gave their written informed consent before study inclusion.

### 2.2 Measurements

Information on demographics, medical history, and daily medication use was collected through use of a detailed questionnaire during hospitalization. All laboratory parameters were analyzed immediately after collection from heparinized plasma samples at the central laboratory of the hospital. Hypertension was defined as at least 2 blood pressure recordings >140/90 mmHg and/or use of antihypertensive drugs. Diabetes was defined as symptoms of diabetes and a casual plasma glucose >200 mg/dL (11.1 mmol/L), fasting plasma glucose >126 mg/dL (7.0 mmol/L), 2-hour plasma glucose concentration >200 mg/dL (11.1 mmol/L) from a 75-g oral glucose tolerance test, and/or use of antidiabetic drugs. Dyslipidemia was defined as a fasting serum total cholesterol >200 mg/dL (5.17 mmol/L), low-density lipoprotein cholesterol >130 mg/dL (3.36 mmol/L), triglyceride >150 mg/dL (1.69 mmol/L), high-density lipoprotein cholesterol <40 mg/dL (1.03 mmol/L), and/or chronic use of lipid-lowering drugs. Chronic kidney disease (CKD) was defined as CrCl <60 mL/min. CrCl was calculated using the Cockcroft and Gault formula.^[18]^ Patients with vascular diseases related to the aorta and other arteries—not including coronaries—that were accompanied by excise-related intermittent claudication, revascularization surgery, reduced or absent pulsation, angiographic stenosis of >50%, or combinations of these characteristics were categorized as having peripheral artery disease (PAD). Patients with previous ischemic stroke or transient ischemic attack were defined as having a cerebrovascular accident. The severity of coronary artery disease (CAD) was classified into 1-, 2-, and 3-vessel or left main (LM) diseases. Echocardiography was performed upon patient admission and LVEF was obtained using the Simpson method from apical 2- and 4- chamber views according to protocols established by the American Society of Echocardiography.^[19]^

### 2.3 PCI procedure

All patients were pretreated with a loading dose of 300 mg of clopidogrel or 180 mg of ticagrelor, in addition to a loading dose of 300 mg of aspirin before intervention, unless they had already received antiplatelet drugs. If patients were contraindicated or allergic to aspirin, they were recommended to take cilostazol (200 mg loading dose) instead of aspirin. Coronary angiography and PCI were performed using standard techniques. The interventional strategy, balloon dilatation, or stent deployment techniques, as well as the selection of a particular balloon or stent, were left to the discretion of the operator in all procedures.

### 2.4 Assessment of AAC

**Figure 1:**
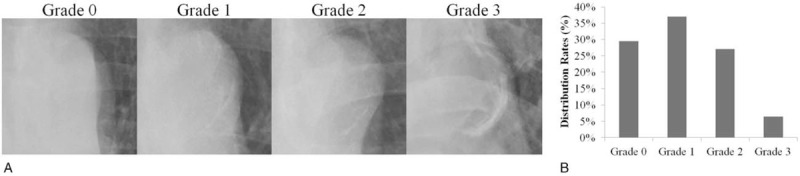
AAC extent across a 4-point scale and distribution of AAC grades. (A) The extent of AAC detected by chest x-ray was divided into 4 grades: Grade 0, no visible calcification (panel A); Grade 1, small spots of calcification or a single thin area of calcification of the aortic knob (panel B); Grade 2, one or more areas of thick Calcification (panel  C); Grade 3, circular calcification of the aortic knob (panel D). (B) Distribution of AAC grades in all patients. AAC = aortic arch calcification.

All patients received either routine posterior-anterior chest X-rays (AXIOM Aristos MX, SIEMENS, Germany) or portable chest x-rays (MUX-200D, SHIMADZU, Japan) on admission. AAC was graded semiquantitatively on a 4-point scale (0–3) (Fig. 1A): grade 0, no visible calcification; grade 1, small spots of calcification or a single thin area of calcification of the aortic knob; grade 2, ≥1 areas of thick calcification; grade 3, circular calcification of the aortic knob.^[20]^ AAC extent was evaluated by 2 independent radiologists who were blinded to other clinical information about the study patients. If disputes arose, the opinion of another more experienced radiologist was obtained and the final decision was made by consensus. Patients were assigned to 4 groups according to AAC extent.

### 2.5 Study end points

Follow-up data were obtained through telephone contact with the patients or their family members at 1 month and every 6 months after discharge using a standardized questionnaire by trained personnel blinded to the AAC extent. Adverse events were ascertained from a careful review of corresponding medical records. The primary end point of the present study was the occurrence of major adverse CV events (MACE), which was defined as a composite of death, nonfatal stroke, nonfatal myocardial infarction (MI), and unplanned repeat revascularization. Death was defined as all-cause mortality. MI was defined as elevated cardiac enzyme levels—such as cardiac troponin and the MB fraction of creatine kinase—which was higher than the upper limit of the normal range and presenting either ischemic symptoms or electrocardiographic changes, implicating ischemia. The presence of new pathological Q waves in ≥2 contiguous electrocardiogram leads was also diagnosed as MI. Within 1 week after PCI, only Q-wave MI was adjudicated as MI. Unplanned repeat revascularization was defined as any nonstaged revascularization after the index PCI. Staged revascularization was defined as scheduled revascularization within 90 days after the index PCI and without treatment of a coronary artery territory that had been treated during the index PCI, or a revascularization status of emergency, urgency, or salvage. Stroke was defined as ischemic cerebral infarction with evidence of neurological dysfunction that required hospitalization for clinically documented lesions on brain CT or magnetic resonance imaging. The key secondary end point was a composite of CV death, nonfatal stroke, or nonfatal MI. Death was considered to be caused by CV unless a definite non-CV cause could be identified. The most severe end point was selected for the primary and key secondary end point analyses if >1 end point occurred during follow- up (death > stroke > MI > revascularization). If >1 stroke, MI, or revascularization occurred, only the first stroke or MI or revascularization was selected.

### 2.6 Statistical analysis

Continuous variables were expressed as the mean ± standard deviation if consistent with a normal distribution, otherwise as the median (0.25–0.75 percentiles). Categorical variables were presented as number and percentage. Interobserver agreement was evaluated by weighted κ statistics, with linear weightings for rating differences; a κ value > 0.81 was considered to denote excellent agreement.^[21]^ To test for differences in continuous variables between groups, Student *t* test or the Mann-Whitney *U* test and analysis of variance or the Kruskal-Wallis H test were applied. For comparison of categorical variables between groups, the χ^2^ test or Fisher exact test was used. To determine factors that were independently related to AAC extent multivariate logistic regression analysis was used. Kaplan–Meier methods were used to derive the event rates at follow-up and to plot time-to-event curves. Differences among Kaplan–Meier estimates of 4 AAC grades were evaluated with the log-rank test. Hazard ratios (HRs) with the corresponding 95% confidence intervals (CIs) for MACE were calculated using Cox-proportional hazards regression analyses. Predictors of MACE identified through univariate analysis were tested in a multivariate Cox-proportional hazards regression analysis. Variables with a univariate significance level ≤.10 that did not cause internal correlations were entered into the multivariate regression model. The validity of the proportionality assumption was verified for all covariates by a visual examination of the log (minus log) curves and a test based on the Schoenfeld residuals. Estimates of the C-index for MACE were calculated after combining the AAC extent to other risk factors associated with independent predictive variables that were identified in the multivariate Cox-proportional hazards regression analyses. The incremental effect of adding the AAC extent to the other risk factors in predicting MACE was evaluated using the net reclassification improvement as previously described.^[22]^ All statistical tests were 2-tailed and *P* <.05 was considered to be statistically significant. Statistical analysis was performed using SPSS 24.0 (IBM, Armonk, NY) and R 3.5.3 (R Foundation for Statistical Computing, Beijing, China).

## 3 Results

The AAC grades of the study patients are shown in Figure 1B. Excellent interobserver agreement of AAC extent was noted, with weighted κ statistics of 0.83 (*P* < .001).

The mean age of the cohort was 59 ± 10 years. Among all patients, 76.8% were male, 65.1% had hypertension, 80.6% had dyslipidemia, 41.7% had diabetes, 5.3% had CKD, 20.0% had a previous MI, 20.0% had a past PCI, 6.0% had a previous cerebrovascular accident, 9.5% had PAD, and 23.8% were diagnosed with acute MI. Baseline characteristics of the study patients based on each AAC grade are shown in Table 1. Patients with higher AAC grades tended to be older, were predominantly female, had a significantly lower rate of current smoking, and were more likely to have more comorbidities, including hypertension, diabetes, CKD, previous MI, past PCI, previous cerebrovascular accident, and/or PAD. In contrast, patients with higher AAC grades were more likely to have higher levels of fasting plasma glucose and glycosylated hemoglobin A1c, as well as increased systolic blood pressure and pulse pressure, while also having lower levels of triglyceride, CrCl, serum calcium, and diastolic blood pressure. Patients with higher AAC grades tended to have lower LVEF and higher levels of brain natriuretic peptide. Details of the procedural information of these patients based on each AAC grade are reported in Table 2. Patients with higher ACC grades were more likely to have higher rates of triple-vessel or LM disease, proximal left anterior descending stenosis, restenotic lesions, and chronic total occlusions.

**Table 1:**
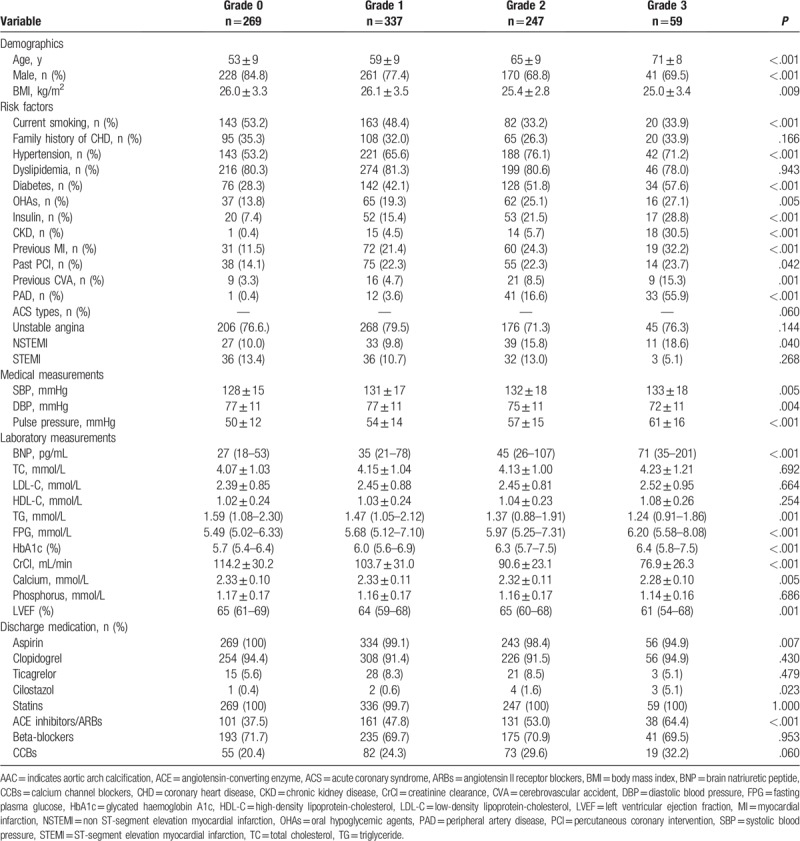
Baseline characteristics of patients according to each AAC grade.

**Table 2:**
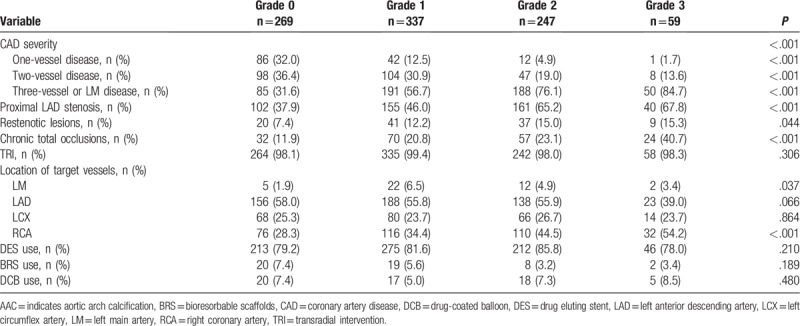
Angiographic findings and interventional characteristics of patients according to each AAC grade.

**Figure 2:**
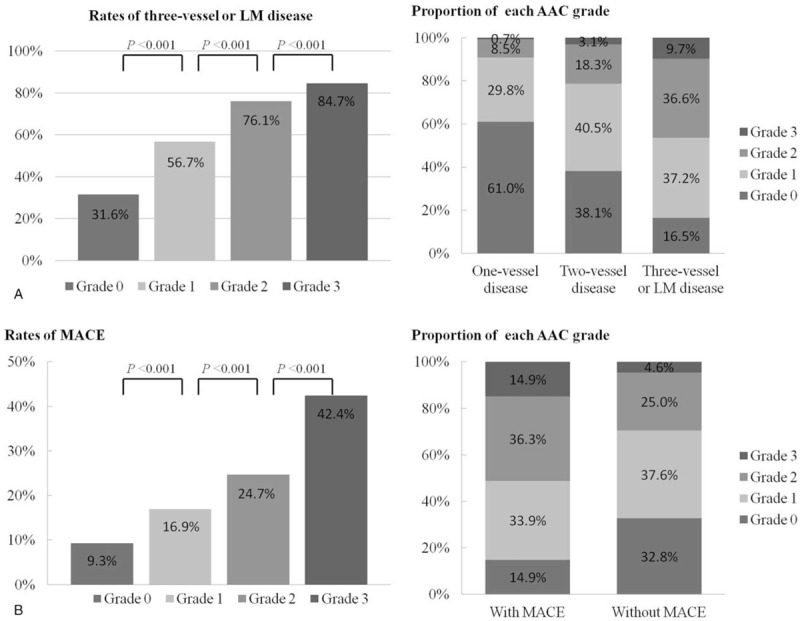
A. AAC grades and CAD severity. Left: rates of triple-vessel or LM disease among patients with AAC grades 0, 1, 2, and 3. Right: proportion of each AAC grade according to CAD severity. B. AAC grades and MACE. Left: rates of MACE among patients with AAC grades 0, 1, 2, and 3. Right: proportion of each AAC grade compared between patients with and without MACE. AAC = indicates aortic arch calcification, CAD = coronary artery disease, LM = left main artery, MACE = major adverse cardiovascular events.

The left side of Figure 2A shows the distribution of triple-vessel or LM disease among AAC grades 0, 1, 2, and 3; the rate of triple-vessel or LM disease positively correlated with higher AAC grade. The proportions of AAC grades ≥2 among 1-, 2-, and 3-vessel or LM diseases were 9.2%, 21.4%, and 46.3%, respectively (*P* < .001; Fig. 2A, right side).

Multivariate regression analysis was performed to determine which variables were independently related to the AAC extent. Statistically significant correlates of AAC extent were age (coefficient β = 0.102, *P* < .001), female (coefficient β = 0.484, *P* = .012), hypertension (coefficient β = 0.414, *P* = .009), diabetes (coefficient β = 0.360, *P* = .012), CKD (coefficient β = 0.681, *P* = .040), PAD (coefficient β = 2.590, *P* < .001), LVEF (coefficient β = 0.023, *P* = .039), 2-vessel disease (compared to 1-vessel disease, coefficient β = 0.975, *P* < .001), 3-vessel or LM disease (compared to 1-vessel disease, coefficient β = 1.984, *P* < .001), proximal left anterior descending stenosis (coefficient β = 1.074, *P* < .001), and chronic total occlusions (coefficient β = 0.738, *P* < .001).

During the follow-up period, MACE occurred in 168 (18.4%) patients, including 22 (2.4%) deaths, 17 (1.9%) deaths from CV causes, 5 (0.5%) deaths from non-CV causes, 11 (1.2%) nonfatal strokes, 28 (3.1%) events of nonfatal MI, and 148 (16.2%) cases of unplanned repeat revascularization.

The clinical outcomes among patients with AAC grades 0, 1, 2, and 3 are shown in Table 3. Compared with those without, patients with MACE had higher rates of diabetes (n = 90, 53.6% vs n = 290, 39.0%; *P* = .001), PAD (n = 38, 22.6% vs n = 49, 6.6%; *P* < .001), previous MI (n = 43, 25.6% vs n = 139, 18.7%; *P* = .043) and past PCI (n = 44, 26.2% vs n = 138, 18.5%; *P* = .025). Patients with MACE had higher pulse pressure (56±15 vs 54±14; *P* = .037), increased levels of fasting plasma glucose (6.14 [5.34–8.07] vs 5.63 [5.10–6.85]; *P* < .001), and higher glycosylated hemoglobin A1c (6.4 [5.7–7.4] vs 5.9 [5.5–6.8]; *P* < .001). Moreover, patients with MACE had a higher incidence of 4-vessel or LM disease (n = 120, 71.4% vs n = 394, 53.0%; *P* < .001), more restenotic lesions (n = 31, 18.5% vs n = 76, 10.2%; *P* = .003), and a lower incidence of 2-vessel (n = 34, 20.2% vs n = 222, 29.8%; *P* = .012) and 1-vessel (n = 14, 8.3% vs n = 128, 17.2%; *P* = .004) diseases. LVEF was significantly lower in patients with MACE (63 [58–68] vs 65 [60–68]%; *P* = .028). Use of medications was not different between patients with and without MACE at discharge, except for aspirin (n = 162, 96.4% vs n = 740, 99.5%; *P* = .003) and cilostazol (n = 5, 3.0% vs n = 5, 0.7%; *P* = .029).

**Table 3:**
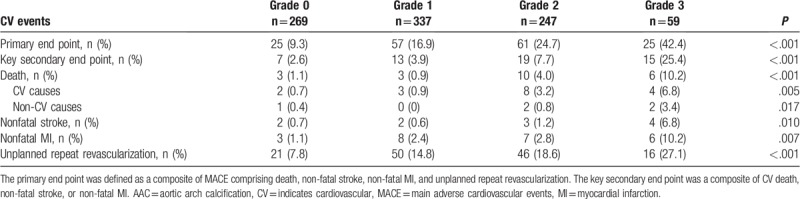
Adverse CV events according to each AAC grade during follow-up.

**Figure 3:**
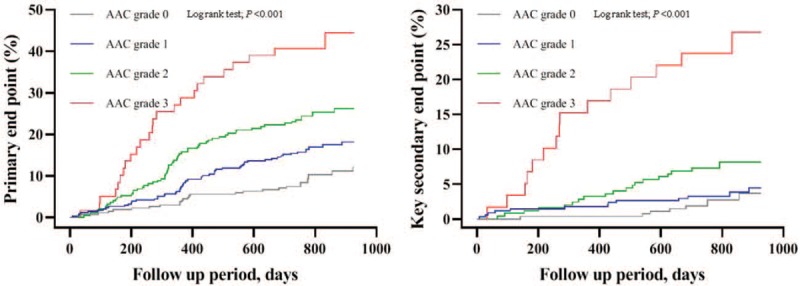
Kaplan-Meier curves for the incidences of the primary and key secondary end points and stratified by AAC grades. The primary end point was defined as a composite of MACE comprising death, nonfatal stroke, nonfatal MI, and unplanned repeat revascularization. The key secondary end point was a composite of CV death, nonfatal stroke, or nonfatal MI. AAC = indicates aortic arch calcification, MACE = major adverse cardiovascular events, MI = myocardial infarction.

The rates of MACE among AAC grades 0, 1, 2, and 3 were 9.3%, 16.9%, 24.7%, and 42.4%, respectively (*P* < .001; Fig. 2B, left side). Patients with MACE had a higher proportion of AAC grades ≥2 than those without MACE (n = 86, 51.2% vs n = 220, 29.6%; *P* < .001; Fig. 2B, right side). Kaplan-Meier analyses revealed significantly higher incidences of primary and key secondary end points in patients with higher AAC grades (log-rank test, all *P* < .001; Fig. 3). Similarly, the incidence of death (log-rank test, *P* < .001), CV death (log-rank test, *P* = .003), nonfatal stroke (log-rank test, *P* = .001), nonfatal MI (log-rank test, *P* = .001), or unplanned repeat revascularization (log-rank test, *P* < .001) was significantly higher in patients with AAC grade 3 than in those with AAC grades 0, 1, or 2.

The results of Cox-proportional hazards regression analyses are shown in Table 4, which includes the AAC extent, diabetes, CKD, previous MI, past PCI, PAD, pulse pressure, serum levels of triglyceride, LVEF, and CAD severity. Multivariate Cox-proportional hazards regression analyses revealed that, in comparison to AAC grade 0, the HRs for AAC grades 1, 2, and 3 in predicting MACE were 1.63 (95% CI 0.99–2.67; *P* = .056), 2.15 (95% CI 1.27–3.62; *P* = .004), and 2.88 (95% CI 1.41–5.86; *P* = .004), respectively.

**Table 4:**
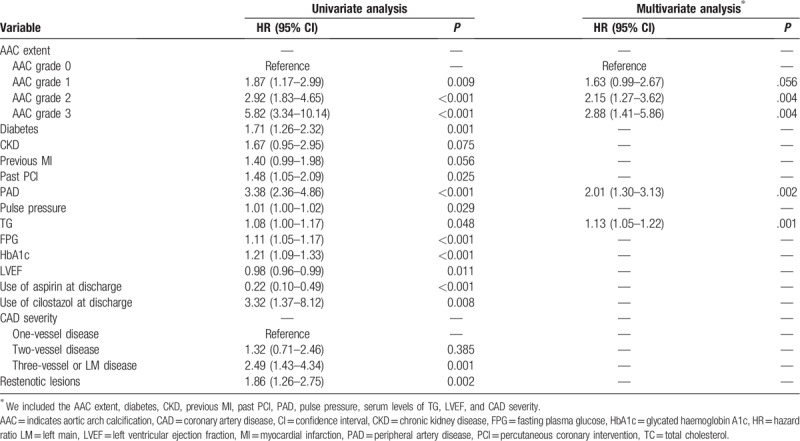
Uni- and multivariate Cox-proportional hazards regression analyses for the prediction of MACE.

Based on the Cox-proportional hazards regression analyses, we calculated the C-index for the predictive value of MACE. The C-index of the variables, including PAD and serum levels of triglyceride, was 0.644 (95% CI 0.600–0.687) versus 0.677 (95% CI 0.635–0.719) when AAC grades were included; the continuous net reclassification improvement was 16.5% (8.7%–23.4%; *P* < .001).

## 4 Discussion

This study revealed 3 important aspects. First, increasing AAC grade is associated with increased risk of MACE among ACS patients undergoing PCI, as determined via log-rank test. Second, multivariate Cox-proportional hazards regression analyses indicated that AAC extent is an independent predictor of MACE in patients with ACS that undergo PCI. Third, predictive modeling is significantly improved after adding AAC extent to the model, as well as including the other independent predictive values. These findings suggest that ACS patients who undergo PCI and have higher AAC grades should receive closer follow-up and more intensive medical therapy.

AAC is associated with CV morbidity and mortality in the general population and several patient cohorts.^[3,9,11–15]^ Iribarren et al^[3]^ evaluated risk factors for AAC and long-term (median follow-up, 28 years) association between AAC and CV diseases in a large population-based cohort study. Among the 116,309 participants, AAC was present in 2.3% of all participants and was independently associated with older age, current smoking, hypertension, and elevated serum cholesterol level. The crude rates (per 1000 person-years) of coronary heart disease (CHD) and ischemic stroke were higher in AAC patients than in those without AAC. After adjustment for several traditional risk factors, AAC was associated with a 1.27-fold increased risk of CHD in men (95% CI, 1.11–1.45), a 1.22-fold increased risk of CHD in women (95% CI 1.07–1.38), and a 1.46-fold increased risk of ischemic stroke in women (95% CI 1.28–1.67). However, the study merely evaluated whether AAC was present or absent using chest x-rays without considering the extent of calcification.

Iijima et al^[9]^ evaluated the validity by which AAC extent can predict new CV events that comprise CHD (angina pectoris, MI), cerebrovascular disease (transient ischemic attack, ischemic stroke, cerebral hemorrhage), PAD, heart failure, and CV death in a retrospective cohort study. AAC was graded according to the same algorithm used in this study. Among 239 asymptomatic outpatients without history of CV events, follow-up recording of CV events was completed with 209 patients. At baseline, the AAC grade was positively related to age, pulse pressure, diabetes, and renal dysfunction. A total of 57 CV events occurred during a mean follow-up period of 69 ± 45 months. Patients with higher AAC grade (grades 2 and 3) had a higher incidence of CV events than those with grade 0 or 1 (*P* < .01). After adjustment for several traditional risk factors, AAC that was detected by chest x-ray was a strong independent predictor of CV events (HR, 2.49; *P* = .01).

A recent meta-analysis was conducted to assess the association between the presence and extent of AAC and CV or all-cause mortality risk in maintenance dialysis patients.^[13]^ A total of 8 observational studies with 3256 dialysis patients were identified, with follow-up duration ranging from 1.8 to 10 years. Compared with patients without AAC, the presence of AAC was associated with greater risk of CV mortality (HR 2.30; 95% CI 1.78–2.97) and all-cause mortality (HR 1.44; 95% CI 1.19–1.75). Subgroup analyses indicated that the pooled HR of AAC grades ≥2 for all-cause mortality was 1.45 (95% CI 1.08–1.96) and CV mortality was 2.31 (95% CI 1.57–3.40).

Recently, the prognostic value of AAC extent for future CV outcomes in patients with stable angina was studied in a large, respective cohort study.^[14]^ Among 2018 patients, 620 had a significant CAD that required coronary revascularization, whereas 191 developed adverse CV events comprising death from all causes, MI, repeated coronary revascularizations, or stroke over a mean follow-up period of 3.8 ± 0.7 years (range 0.7–5.1 years). There were higher rates of significant CAD (Grade 0 vs Grade 1/2 vs Grade 3: 25.9% vs 42.4% vs 54.5%, *P* < .001) and adverse CV events (Grade 0 vs Grade 1/2 vs Grade 3: 8.4% vs 11.1% vs 19.3%, *P* < .001) with increasing AAC grade.

To date, few clinical studies have focused on the prognostic role of AAC extent in patients with ACS that undergo PCI. Yang et al^[15]^ revealed a significant association between AAC extent and outcomes in patients with ACS. Among 225 patients, 190 underwent coronary revascularization at baseline and patients with AAC had a similar revascularization rate to those without AAC (83% vs 87%, *P* = .46), whereas patients with AAC had significantly higher 30-day mortality (17.3% vs 7.1%, log-rank *P* = .02). During a mean follow-up period of 165 ± 140 days (maximum 492 days), patients with AAC had significantly increased CV deaths (27.6% vs 11.2%, log-rank *P* = .002), all-cause mortality (28.3% vs 11.2%, log-rank *P* = .001), and a composite end point of major adverse CV events that comprised nonfatal MI, nonfatal stroke, and CV death (39.4% vs 24.6%, log-rank *P* = .01). However, the study had several major limitations. First, the study was based on a retrospective observational ACS registry. Second, patient characterizations and procedures (ie, revascularization strategy, end point event definition, and follow-up method) were poorly defined. Third, the study had a relatively small sample size and a short follow-up period. Finally, the authors did not consider ACC extent when multivariate Cox-proportional hazards regression analyses were performed to identify AAC as an independent prognostic factor.

There are several advantages and differences within this study as compared to the report by Yang et al. This report is based on a prospective study that had a larger sample size and a longer follow-up period. Moreover, this study described patient baseline characteristics in more detail and better-defined end point events and follow-up methods. In addition, this study used AAC grades as the basis for grouping, demonstrating a potent predictive value of using high AAC grades to precisely forecast subsequent adverse CV events. Analyzing HRs for each AAC grade, AAC grade 1 had a borderline significance (*P* = .056) for predicting MACE compared to grade 0, suggesting that even trivial calcium deposition in aortic arch implies increased CV risk for ACS patients undergoing PCI.

Aortic calcification is closely associated with increased aortic stiffness,^[23–25]^ often resulting in early wave reflection of the aortic pulse wave. Consequently, the early pulse wave reflection increases systolic blood pressure but decreases diastolic blood pressure. These hemodynamic changes cause increased left ventricular afterload and myocardial wall stress, as well as impair coronary perfusion.^[26]^ Furthermore, several studies have indicated that increased aortic stiffness is an independent predictor of adverse CV events in patients with acute MI.^[26,27]^ Aortic calcification is significantly associated with vascular endothelial dysfunction,^[9]^ which generally triggers platelet adhesion and aggregation, in addition to fibrin formation, which all play critical roles in systemic hypercoagulability.^[28]^ Adverse CV events, such as MI^[29]^ or ischemic stroke,^[30]^ have been demonstrated to be commonly characterized at the pathophysiological level by vascular endothelial dysfunction. Moreover, vascular endothelial dysfunction is a vital component of both coronary plaque vulnerability and other CV complications, such as vascular remodeling.^[31]^ Aortic calcification is also associated with decreased coronary flow reserve as measured by ^82^Rb positron emission tomography/computed tomography,^[32]^ which is positively correlated with CV morbidity and mortality. Notably, a previous study revealed that AAC observed by chest x-ray or fluoroscopy is significantly associated with greater necrotic core-containing plaques—as detected by virtual histology and intravascular ultrasound—which is an independent predictor of adverse CV events.^[33]^ The aforementioned sequential associations suggest a potential prognostic relevance for AAC in ACS patients.

Older age, female sex, hypertension, diabetes, CKD, PAD, LVEF, and CAD severity are all important risk factors for future CV events in ACS patients. In this study, we found that AAC extent was significantly correlated with these risk factors, which also partially explains the association between AAC extent and adverse CV outcomes. We demonstrated that increasing AAC grade was independently associated with higher rates of MACE, even after adjusting for diabetes, CKD, previous MI, past PCI, PAD, pulse pressure, serum levels of triglyceride, LVEF, and CAD severity, while adding AAC extent to a risk model that is comprised of other independent MACE predictors significantly improved the early risk stratification of ACS patients treated with PCI. These findings are of significant clinical relevance, as assessing AAC extent might contribute to improved early risk stratification for ACS patients undergoing PCI, which could enhance prognostic evaluations and guidance of secondary prevention treatment. Patients who develop MACE are older and exhibit higher incidences of coronary triple-vessel or LM disease, highlighting the prognostic role for these conditions.

### 4.1 Limitations

There are several limitations in the present study. First, as in any observational study, this study cannot exclude influences that were due to unmeasured and undetected confounding variables such as calcification-related biomarkers, SYNTAX Score, periprocedural anticoagulants use, and drug eluting stent types. However, we used as many well-known risk factors as possible for CV outcomes as confounders. Second, at each follow-up, we recorded the medications the patients were taking. Medication adjustment according to patients’ conditions was frequent, especially year after PCI. As we know, the change of medications was also associated with MACE. However, we did not include medication adherence in the analysis. Third, chest x-ray-based methods to assess AAC are only semiquantitative and the precise amount of calcium deposition in the aortic arch could be underestimated. Fourth, positional changes on chest x-rays could potentially alter the appearance of AAC and influence the measured value of AAC thickness. Fifth, follow-up data were obtained via telephone, but the authenticity of adverse events was often verified by obtaining corresponding medical records from patients or their family members. Finally, the patients enrolled in this study were in relatively stable condition. Patients who were unstable on admission and those who developed severe heart failure (LVEF <30% or Killip class >2) were not included. Therefore, the results of this study cannot be generalized to other ACS patient cohorts, particularly those that include patients with unstable hemodynamic conditions. Moreover, all patients in this study had been treated with PCI and, therefore, our results may not be applicable to patients undergoing coronary artery bypass grafting or were treated conservatively.

## 5 Conclusions

In this well-designed prospective study, the extent of AAC as detected by chest x-ray was demonstrated to be an independent predictor of MACE in ACS patients undergoing PCI at a mean follow-up of 917 days. Further research is required to evaluate whether specific treatment strategies based on AAC extent are useful for optimal risk reduction in relevant patient populations.

## Acknowledgments

The authors thank LetPub (www.letpub.com) for linguistic assistance during the revision of this manuscript.

## Author contributions

**Conceptualization:** Zhijian Wang, Yujie Zhou.

**Data curation:** Lixia Yang, Zhijian Wang, Yujie Zhou.

**Formal analysis:** Xiaoteng Ma, Lisha Dong, Zhen Zhou, Jing Tian, Zhijian Wang, Yujie Zhou.

**Funding acquisition:** Yujie Zhou.

**Investigation:** Xiaoteng Ma, Lisha Dong, Qiaoyu Shao, Zhen Zhou, Jing Tian, Yue Ma, Jie Yang, Sai Lv, Yujing Cheng, Hua Shen.

**Methodology:** Xiaoteng Ma, Lixia Yang, Zhijian Wang, Yujie Zhou.

**Project administration:** Yujie Zhou.

**Resources:** Yujie Zhou.

**Software:** Xiaoteng Ma, Lisha Dong, Qiaoyu Shao, Lixia Yang, Zhijian Wang.

**Supervision:** Zhijian Wang, Yujie Zhou.

**Validation:** Hua Shen, Lixia Yang, Zhijian Wang, Yujie Zhou.

**Visualization:** Zhijian Wang, Yujie Zhou.

**Writing – original draft:** Xiaoteng Ma, Lisha Dong.

**Writing – review & editing:** Zhijian Wang, Yujie Zhou.
